# An eDNA/eRNA‐based approach to investigate the life cycle of non‐cultivable shellfish micro‐parasites: the case of *Bonamia ostreae*, a parasite of the European flat oyster *Ostrea edulis*

**DOI:** 10.1111/1751-7915.13617

**Published:** 2020-07-01

**Authors:** Nicolas Mérou, Cyrielle Lecadet, Stéphane Pouvreau, Isabelle Arzul

**Affiliations:** ^1^ Laboratoire de Génétique et Pathologie des Mollusques Marins Ifremer SG2M‐LGPMM Avenue de Mus de Loup 17390 La Tremblade France; ^2^ Laboratoire des Sciences de l'Environnement Marin UMR 6539, Ifremer/UBO/IRD/CNRS Ifremer 11 Presqu'île du Vivier 29840 Argenton‐en‐Landunvez France

## Abstract

Environmental DNA approaches are increasingly used to detect microorganisms in environmental compartments, including water. They show considerable advantages to study non‐cultivable microorganisms like *Bonamia ostreae,* a protozoan parasite inducing significant mortality in populations of flat oyster *Ostrea edulis*. Although *B. ostreae* development within the host has been well described, questions remain about its behaviour in the environment. As *B. ostreae* transmission is direct, seawater appears as an interesting target to develop early detection tools and improve our understanding of disease transmission mechanisms. In this context, we have developed an eDNA/eRNA approach allowing detecting and quantifying *B. ostreae* 18S rDNA/rRNA as well as monitoring its presence in seawater by real‐time PCR. *B. ostreae* DNA could be detected up to 4 days while RNA could be detected up to 30 days, suggesting a higher sensitivity of the eRNA‐based tool. Additionally, more than 90% of shed parasites were no longer detected after 2 days outside the oysters. By allowing *B. ostreae* detection in seawater, this approach would not only be useful to monitor the presence of the parasite in oyster production areas but also to evaluate the effect of changing environmental factors on parasite survival and transmission.

## Graphical abstract

An eDNA/eRNA approach was developed, allowing detecting and quantifying *Bonamia ostreae* 18S rDNA/rRNA as well as monitoring its presence in seawater by real‐time PCR. *B. ostreae* DNA and RNA could be detected up to 4 and 30 days respectively, suggesting a higher sensitivity of the eRNA based tool. More than 90% of shed parasites were no longer detected after 2 days outside the oysters.

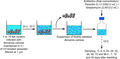

## Introduction

The flat oyster *Ostrea edulis* (Linnaeus, 1758) is a bivalve species native to Europe. The history of its production has been affected by overfishing, environmental degradation and several disease outbreaks (Buestel *et al.*, 2009), including the emergence of bonamiosis in 1979, an epizootic disease caused by the protozoan parasite *Bonamia ostreae*. This parasite was first described in Ile Tudy (Brittany, France) in the context of flat oyster mortality (Pichot *et al.*, 1980) and has since spread around Europe, Canada (British Columbia) and United States of America (California, Maine and Washington States) (OIE, 2020), mainly through aquaculture‐related movements (Pogoda *et al.*, 2019). More recently, it has also been reported in *Ostrea chilensis* in New Zealand (Lane *et al.*, 2016). Today, bonamiosis still causes severe losses, particularly in natural populations, that might hamper the different projects of flat oyster restoration currently undertaken in several European countries (Pogoda *et al.*, 2019; NORA, 2020). Considering its impact on natural and farmed bivalves, *B. ostreae* is notifiable to the World Organisation for Animal Health and the European Union.

Oysters infected with *B. ostreae* can be found in various ecosystems, from estuaries and intertidal zones to deep coastal waters or lagoons. All life stages of the flat oyster including larvae, spat and adults can be infected by the parasite. However, mortalities mainly affect flat oysters older than 2 years (Culloty and Mulcahy, 1996). New oysters can become infected with *B. ostreae* throughout the year but prevalence and infection intensity within flat oyster usually show a peak at the end of winter‐early spring (Culloty and Mulcahy, 1996; Arzul *et al.*, 2006; Engelsma *et al.*, 2010).

*Bonamia ostreae* belongs to the *Haplosporida* order. It multiplies within haemocyte cytoplasm where it can be observed under light microscopy as small rounded cells measuring between 2 and 5 µm in diameter. Sometimes, the parasite is present extracellularly. Its presence is often associated with intense haemocyte infiltration (Arzul and Carnegie, 2015). Based on an electron microscopy study, a possible life cycle of *B. ostreae* in which the parasite is suspected to enter and leave its host through gills has been suggested (Montes *et al.*, 1994). This hypothesis is supported by the observation of parasites in gill epithelium of highly infected oysters by histology and *in situ* hybridization observation (Arzul and Carnegie, 2015).

*Bonamia ostreae* is not cultivable; however, a protocol allows isolating parasites from highly infected oysters (Mialhe *et al.*, 1988). It is possible to experimentally infect oysters by injecting parasites isolated following this protocol in the adductor muscle or the pericardic cavity of the flat oyster (Hervio *et al.*, 1995). Additionally, it is possible to infect oysters by maintaining them in cohabitation with infected oysters, demonstrating that the parasite can be directly transmitted and does not require intermediate host to complete its life cycle (Culloty *et al.*, 1999; Lallias *et al.*, 2008).

As many other shellfish diseases, bonamiosis is a non‐pathognomonic disease and its diagnosis relies on cytology, histology and PCR‐based approaches. Several PCR assays have been developed allowing the detection of *B. ostreae* DNA in flat oysters including some conventional (Carnegie *et al.*, 2000; Cochennec *et al.*, 2000) and, more recently, some real‐time PCR tools (Robert *et al.*, 2009; Ramilo *et al.*, 2013). The surveillance of the parasite is carried out by testing flat oysters as recommended by the OIE (OIE, 2020) whereas its detection outside *Ostrea edulis* had only been investigated for research purposes.

As *B. ostreae* is suggested to be directly transmitted through water column (Arzul and Carnegie, 2015), understanding its life cycle requires approaches allowing the detection of the parasite and monitoring its presence in seawater. Considering that *B. ostreae* is not cultivable, environmental DNA (eDNA)‐based approach appears of interest to monitor its presence in seawater. Although presenting some methodological constraints particularly in aquatic environments, eDNA analyses allow rapid, non‐invasive and cost‐efficient biodiversity monitoring (Harper *et al.*, 2019). Most of the currently developed pathogen eDNA studies target bacteria and more particularly human pathogens (Green *et al.*, 2011; Twing *et al.*, 2011; Casanovas‐Massana *et al.*, 2018) but some work have been carried out on marine and freshwater pathogens (Goarant and Merien, 2006; Johnson and Brunner, 2014; Strepparava *et al.*, 2014; Rusch *et al.*, 2018). In the context of shellfish diseases, eDNA studies have revealed new parasite clades and parasite diversity (Ward *et al.*, 2016; Ward *et al.*, 2018), but also to investigate pathogen survival outside its host (Eiler *et al.*, 2006; Friedman *et al.*, 2014) or the infection process (Parizadeh *et al.*, 2018). Nevertheless, DNA detection does not allow discriminating between viable and non‐viable cells. In this context, it can be interesting to focus on RNA detection, as it is suggested to be a better proxy for the presence of metabolically active stages, due to its rapid degradation in the environment (McCarthy *et al.*, 2001; Nocker *et al.*, 2007; Bae and Wuertz, 2009; Pochon *et al.*, 2017). Indeed, being able to discriminate viable and non‐viable cells is fundamental, especially where parasite life cycles are poorly known such as *B. ostreae* cycle. However, recent studies also suggest that RNA can persist in the environment through the protection within extracellular vesicles (Cristescu, 2019). RNA may not allow a strict distinction between viable and non‐viable cells, that is why it could be preferable to associate both DNA and RNA detection in environmental studies.

In this context, we have developed and combined real‐time PCR‐based approaches to detect *B. ostreae* 18S rDNA and rRNA in seawater. These tools are useful for detecting and quantifying the parasite in seawater but can also be used to better understand parasite life cycle outside its host.

## Results

### Real‐time PCR efficiency, limit of detection, limit of quantification and standard curves

#### *Bonamia ostreae* DNA detection

The efficiency, limit of detection (LOD) and limit of quantification (LOQ) of the *Bonamia ostreae* real‐time PCR detection approach were determined from DNA extracted from five 10‐fold dilution series of 1 µm polycarbonate membranes artificially contaminated with 2.5 to 2.5 x 10^5^ purified parasites (per quarter membrane) (see Experimental procedures section for further details).

Real‐time PCR efficiency was 93.47 % (SD = 4.35), and the analysis of parasite dilution series allowed the detection of *B. ostreae* DNA from 2.5 to 2.5 × 10^5^ parasites (per quarter membrane). However, the lowest tested parasite amount was only detected twice out of the five tests. Considering that the detection limit was between 2.5 and 25 parasites per quarter membrane, DNA extracted from this last condition was diluted in order to get the following equivalent parasite quantities: 15, 7.5 and 2.5 parasites. These dilutions were tested six times. It was possible to detect 15 parasites in the six replicates, 7.5 parasites in five replicates and 2.5 parasites in one replicate. The LOD of the DNA‐based approach was set at 7.5 parasites per quarter membrane as it represents more than 75% of the tested replicates.

The LOQ of the *B. ostreae* DNA detection approach was also set at 7.5 parasites as it was the last amount of detected parasites in the linear dynamic range of the standard curve.

**Fig. 1:**
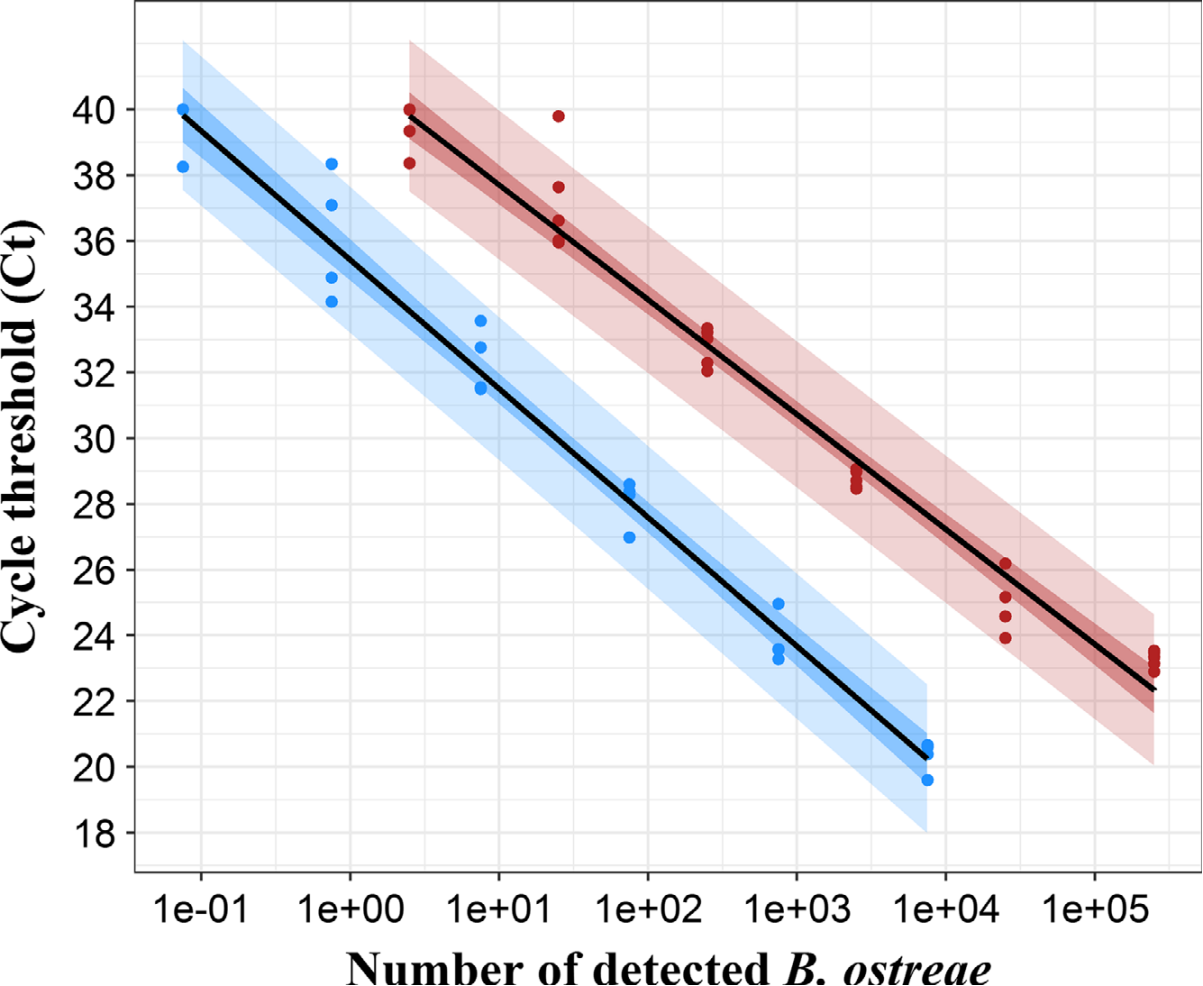
Standard curves established from DNA (red) and RNA (blue) real‐time PCR analyses of *Bonamia ostreae* dilution series. *Ct*_DNA_ = −1.52 × ln(*N*_parasites_) + 41.2, *R* ^2^ = 0.97; *Ct*_RNA_ = −1.7 × ln(*N*_parasites_) + 35.42, *R* ^2^ = 0.98. Dots represent experimental data, solid lines represent linear regressions and coloured areas represent 95% confidence (dark) and 95% prediction (light) intervals associated with each regression. For each parasite amount, *n* = 5 for DNA and *n* = 4 for RNA.

**Fig. 2:**
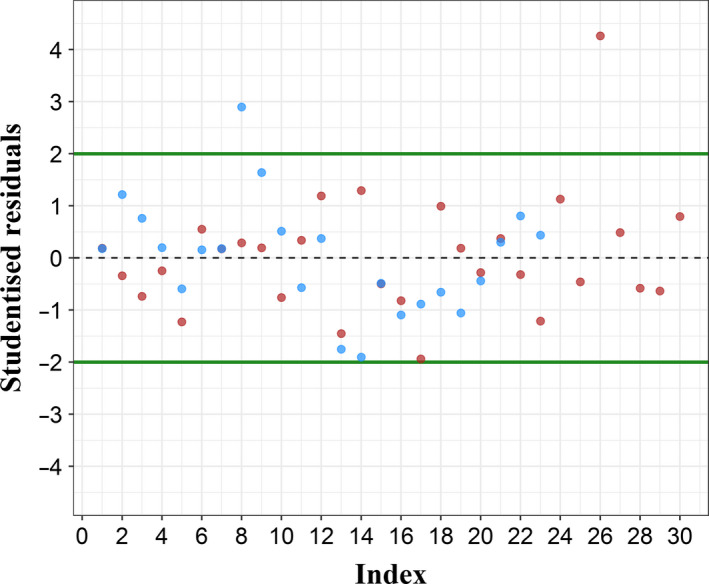
Studentized residuals associated with each regression. Red dots represent studentized residuals associated with DNA standard curve (*n* = 30). Blue dots represent studentized residuals associated with RNA standard curve (*n* = 24).

A total of 30 measures could be associated with cycle threshold (*Ct*) and the number of detected parasites (*N*_parasites_). The data were thus considered as normally distributed. The following linear regression was performed on *Ct* values obtained by real‐time PCR and logarithm of number of detected parasites, ln(*N*_parasites_): *Ct*_DNA_ = −1.52 × ln(*N*_parasites_) + 41.2 (Fig. 1). This regression was associated to a high *R* ^2^ = 0.97 meaning that 97% of the variability on the *Ct* can be explained by the *N*_parasites_. Furthermore, the analysis of studentized residuals (Fig. 2 red dots) allowed validating the linear regression. These residuals were homogeneously and continuously distributed around 0 and more than 95% of them (29 over 30) were between −2 and 2. The prediction interval included all except one point, which was close to the limit of detection of the approach.

#### *Bonamia ostreae* RNA detection

The efficiency, LOD and LOQ of the *Bonamia ostreae* RNA detection approach were determined by analysing by reverse transcription real‐time PCR RNA extracted from four 10‐fold dilution series of 1 µm polycarbonate membranes artificially contaminated with 7.5 × 10^−2^ to 7.5 × 10^6^ purified parasites (per quarter membrane) (see Experimental procedures section for further details).

Real‐time PCR efficiency was 81.96 % (SD = 9.07), and the analysis of parasite dilution series allowed the detection of *B. ostreae* RNA from 7.5 × 10^−2^ to 7.5 × 10^3^ parasites (per quarter membrane). The lowest tested parasite amount was only detected once whereas the one with 0.75 parasites was detected four times out of four. Thereby, the LOD of the RNA‐based approach was set at 0.75 parasites per quarter membrane.

The LOQ of the *B. ostreae* RNA detection approach was also set at 0.75 parasites as it was the last amount of detected parasites in the linear dynamic range of the standard curve. Less than 30 measures (*n* = 24) could be associated with *Ct* and *N*_parasites_. Data normality was checked using Shapiro–Wilk test. *Ct* data were found normally distributed, contrary to *N*_parasites_ data, whereas ln(*N*_parasites_) data were normally distributed. As for the DNA detection‐based approach, the following linear regression was performed on *Ct* and ln(*N*_parasites_): *Ct*_RNA_ = −1.7 × ln(*N*_parasites_) + 35.42 (Fig. 1). This regression was associated with a high *R* ^2^ = 0.98, which means that 98% of the variability on the *Ct* can be explained by the number of detected parasites. The analysis of studentized residuals (Fig. 2, blue dots) allowed validating the linear regression. These residuals were indeed homogeneously and continuously distributed around 0 and more than 95% of them (23 over 24) were between −2 and 2. The prediction interval included all except one experimental point, which was close to the limit of detection of the approach.

### Monitoring of *Bonamia ostreae* presence in seawater

*Bonamia ostreae* presence in seawater was monitored from twelve freshly released parasite suspensions obtained by incubating infected flat oysters in UV‐treated seawater filtered at 1 µm during 24 h. After 24 h, oysters were removed and parasite presence in seawater was monitored until one month by real‐time PCR and reverse transcription real‐time PCR (see Experimental procedures section for further details).

At *t* = 0, depending on the tested parasite suspension, from 4.00 × 10^3^ to 7.02 × 10^5^ parasites and from 1.37 × 10^3^ to 4.30 × 10^4^ parasites were detected per quarter membrane using the DNA‐ and RNA‐based approaches respectively (Table 1). After 96 h, parasite DNA was still detected in 2 of the 3 tested suspensions (3.74 × 10^1^ and 1.06 × 10^3^ parasites), while parasite RNA was still detected in all three tested suspensions (from 6.17 to 6.04 × 10^1^ parasites) (Table 1). After 7 days, parasite DNA was no longer detected, whereas parasite RNA remained detectable in most of the tested suspensions even after 30 days (Table 1). Nevertheless, the amount of detected parasite was at least 100 times lower than the number of parasites detected at the beginning of the experiment.

**Table 1:** Number of detected *Bonamia ostreae* in each tested parasite suspension at different times after removing flat oysters using both DNA and RNA real‐time PCR‐based approaches.

	*t* = 0 day	*t* = 1 day	*t* = 2 days	*t* = 7 days	*t* = 15 days	*t* = 30 days
DNA						
Suspension 1	4.00E + 03	4.20E + 03	7.50E + 00^a^	7.50E + 00^a^	7.50E + 00^a^	7.50E + 00^a^
Suspension 2	1.48E + 05	1.22E + 05	1.21E + 04	7.50E + 00^a^	7.50E + 00^a^	7.50E + 00^a^
Suspension 3	1.54E + 05	1.14E + 05	9.97E + 00	7.50E + 00^a^	7.50E + 00^a^	7.50E + 00^a^
Suspension 4	6.22E + 05	1.03E + 05	1.58E + 04	7.50E + 00^a^	7.50E + 00^a^	7.50E + 00
Suspension 5	3.42E + 05	1.05E + 05	6.08E + 03	7.50E + 00^a^	7.50E + 00^a^	7.50E + 00^a^
Suspension 7	7.02E + 05	2.83E + 04	1.03E + 04	7.50E + 00^a^	7.50E + 00^a^	7.50E + 00^a^
Suspension 8	1.55E + 05	3.13E + 05	2.02E + 04	7.50E + 00^a^	7.50E + 00^a^	7.50E + 00^a^
Suspension 9	1.84E + 05	4.73E + 05	9.10E + 04	7.50E + 00^a^	7.50E + 00^a^	7.50E + 00^a^
RNA						
Suspension 1	1.25E + 04	2.63E + 03	1.37E + 02	8.28E + 00	1.03E + 01	1.27E + 01
Suspension 2	4.60E + 03	2.18E + 04	7.20E + 02	7.50E−01^a^	4.73E + 01	7.50E−01^a^
Suspension 3	8.29E + 03	7.50E−01^a^	2.36E + 02	7.50E−01^a^	3.52E + 01	2.69E + 00
Suspension 4	1.10E + 04	1.16E + 04	4.69E + 02	3.00E + 00	2.07E + 01	4.35E + 00
Suspension 5	1.37E + 03	7.50E−01^a^	3.66E + 01	1.32E + 01	1.39E + 00	7.74E + 00
Suspension 7	4.30E + 04	1.62E + 04	3.25E + 00	7.50E−01^a^	2.38E + 01	7.50E−01^a^
Suspension 8	2.35E + 04	7.50E−01^a^	4.38E + 03	7.50E−01^a^	2.12E + 01	3.84E + 03^b^
Suspension 9	2.30E + 03	1.34E + 04	8.30E + 01	9.23E + 00	7.50E−01^a^	7.50E−01^a^

	*t* = 0 h	*t* = 4 h	*t* = 8 h	*t* = 24 h	*t* = 28 h	*t* = 32 h	*t* = 48 h	*t* = 56 h	*t* = 72 h	*t* = 96 h
DNA										
Suspension 10	6.35E + 03	5.12E + 03	2.55E + 04	7.99E + 03	3.71E + 03	2.10E + 03	6.55E + 02	3.27E + 02	3.01E + 01	3.74E + 01
Suspension 11	2.26E + 04	4.50E + 04	2.24E + 04	2.82E + 04	1.79E + 04	1.79E + 04	8.18E + 03	8.15E + 03	1.72E + 03	7.50E + 00
Suspension 12	2.26E + 04	4.19E + 04	4.71E + 04	1.89E + 04	1.65E + 04	1.10E + 04	7.92E + 03		2.28E + 03	1.06E + 03
RNA										
Suspension 10	4.00E + 02	1.52E + 03	8.42E + 02	1.26E + 03	1.06E + 03	5.95E + 02	1.78E + 02	5.29E + 01	1.11E + 02	6.04E + 01
Suspension 11	5.14E + 02	9.01E + 01	1.40E + 02	2.16E + 02	2.02E + 02	2.46E + 01	4.93E + 01	7.50E−01^a^	5.99E + 01	1.82E + 01
Suspension 12	1.32E + 02	2.24E + 02	1.33E + 01	6.39E + 01	1.51E + 02	6.84E + 01	2.03E + 01		9.91E + 00	6.17E + 00

^a^ Fixed at the limit of detection.

^b^ Considered as outlier and not taken in account in further analysis.

The effect of the initial (*t* = 0) number of parasites on its subsequent detection (*t* = 48 h for the nine first suspensions and *t* = 96 h for the three last suspensions) was tested by computing Spearman’s (*rho*) and Kendall’s (*tau*) correlation coefficients, as data were not normally distributed (Shapiro–Wilk test). Correlation was found positive for both DNA (*rho* = 0.69, *P*‐value = 0.02, and *tau* = 0.53, *P*‐value = 0.03) and RNA detection (*rho* = 0.39, *P*‐value = 0.2, and *tau* = 0.38, *P*‐value = 0.12), but only marginally significant in the case of DNA.

**Fig. 3:**
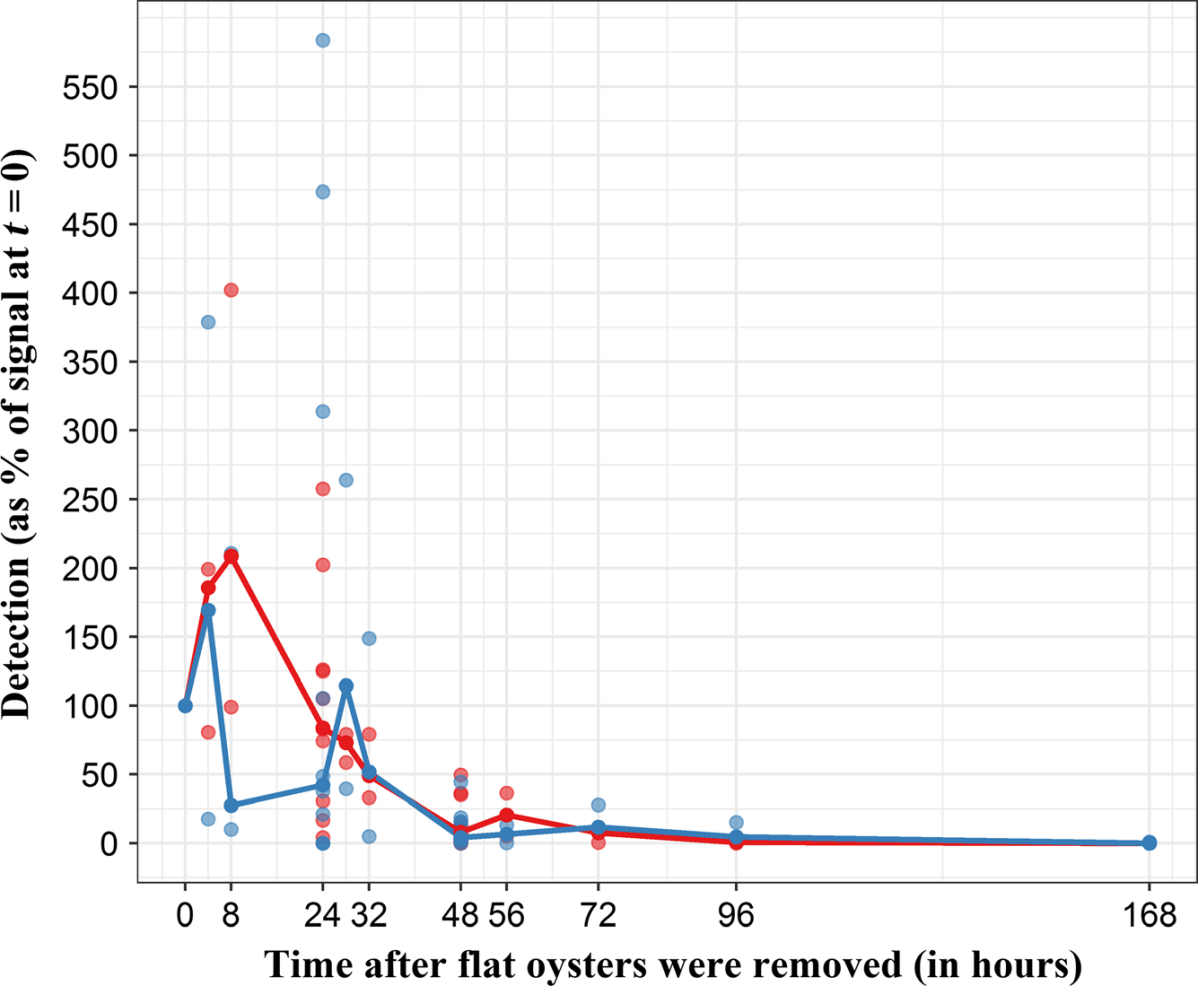
Temporal trend of *Bonamia ostreae* DNA (red) and RNA (blue) detection after removing flat oysters. Detection on *Y*‐axis is computed for each sampling time as the ratio between the number of detected parasites at *t* = *τ* and the number of detected parasites at *t* = 0. Dots represent experimental data, and solid lines represent the median detection trend. For 0, 24, 48 and 168 h, *n* = 11. For other sampling times, *n* = 3. In order to facilitate reading, *t* = 168 h is the last experimental data showed on the graph, considering that number of detected parasites at *t* = 360 h and *t* = 720 h were almost the same.

Median percentages were then computed for each time and for both DNA and RNA detection (Fig. 3, solid lines).

Median DNA detection increased from *t* = 0 to *t* = 8 h (209% of the initial detection) and then decreased to less than 1% of the initial detection at *t* = 168 h (7 days). From 28 h, the amount of detected parasites became statistically different from *t* = 0 (*P*‐value_0−28_ = 5.27 × 10^−4^, Wilcoxon–Mann–Whitney): at 28 h, 73% of the initial parasite quantity was still detected. At *t* = 48 h, only 8% of the initial amount of parasites remained detectable (*P*‐value_0−48_ = 2.55 × 10^−5^, Wilcoxon–Mann–Whitney).

Median RNA detection increased from *t* = 0 to *t* = 4 h (169% of the initial detection), decreased between *t* = 4 h and *t* = 8 h (27% of the initial detection) before increasing again from *t* = 8 h to *t* = 28 h (114% of the initial detection). Finally, after 28 h it decreased up to about 5% of the initial detection. After 48 h, the amount of detected parasites became statistically different from *t* = 0 (*P*‐value_0−48_ = 2.55 × 10^−5^, Wilcoxon–Mann–Whitney). At this time, 6% of the initial signal remained detectable.

*Bonamia ostreae* DNA and RNA detections showed the same evolution over time (Fig. 3). As data were considered as normally distributed (*n* > 30), correlation between DNA and RNA detection was investigated by computing Pearson’s coefficients. Significantly positive correlation was found (*cor* = 0.53; *P*‐value = 4.87 × 10^−7^ and *cor* = 0.67; *P*‐value = 2.08 × 10^−11^) between DNA and RNA when considering detection percentages and *N*_parasites_ respectively.

## Discussion

Studies and monitoring of shellfish diseases are mostly based on pathogen detection within their hosts (Barbosa Solomieu *et al.*, 2015). Focusing on pathogens inside their bivalve hosts maximizes the chance to detect them. However, understanding pathogen life cycles and epizootiology is also needed to better monitor and manage associated diseases. In this context, environmental DNA (eDNA)‐based approaches could be very useful as they present considerable advantages to study pathogens outside their host, notably the detection of elusive or non‐cultivable organisms (Taberlet *et al.*, 2012; Bass *et al.*, 2015) like *Bonamia ostreae*.

Therefore, we have developed for the first time an eDNA/eRNA‐based approach allowing *B. ostreae* detection and quantification in seawater and then used it to monitor parasite presence in seawater until one month after being released.

*Bonamia ostreae* detection was carried out by real‐time and reverse transcription real‐time PCR amplifying the 18S rDNA and rRNA, respectively, from nucleic acid extracted from water after filtration on a 1 µm pore size membrane. Quantification has been possible thanks to standard curves established from serially diluted parasite suspensions and real‐time PCR targeting *B. ostreae* DNA has allowed detecting and quantifying from 7.5 to 2.5 × 10^5^ parasites. The assay targeting RNA has allowed detecting and quantifying from 0.75 to 7.5 × 10^3^ parasites.

Few real‐time PCR assays are currently available to quantify bivalve pathogens and most of them target pathogens in bivalve tissues. For example, a real‐time PCR assay was developed, allowing the quantification of *B. ostreae* DNA from gill tissues with a 6 log dynamic range and a limit of detection of 50 gene copies per reaction (Robert *et al.*, 2009). A similar approach was developed to detect and quantify the protozoan *Perkinsus marinus* parasite of the American oyster *Crassostrea virginica*, not only in oyster tissues but also in environmental waters (Audemard *et al.*, 2004). This DNA‐based approach allowed detecting as low as 3.3 × 10^−2^ cell per 10 µl reaction mixture.

*Bonamia ostreae* RNA detection limit appeared 10 times lower than DNA one, indicating a higher sensitivity of the RNA‐based approach. While the gene encoding 18S RNA might be present in several copies in the parasite genome, RNA and more particularly ribosomal RNA are expected to be much more abundant, depending notably on the metabolism of the cells (Roberts *et al.*, 2011; Ryan *et al.*, 2012).

As *B. ostreae* is not cultivable, contaminated seawater suspensions were obtained by maintaining infected flat oysters in UV‐treated seawater. The use of both RNA‐ and DNA‐based real‐time PCR assays has allowed showing that live infected flat oysters were able to shed parasites. This was suggested (Arzul and Carnegie, 2015) but never demonstrated before, most authors assuming that parasites are released from dying or dead infected oysters.

In our conditions, parasite RNA remained detectable up to 30 days while DNA was no longer detected after 7 days spent outside the host. This difference can be explained by the limit of detection of both approaches. As the limit of detection of the RNA‐based PCR assay is 10 times lower than the DNA approach, smaller parasites quantities can be detected using the RNA assay. This observation is also supported by some recent studies suggesting that, contrary to common thinking, eRNA is abundant and persistent in terrestrial and aquatic environment (Cristescu, 2019). Nevertheless, parasite RNA detected 7, 15 and 30 days post‐shedding always represented less than 1% of the initial detected quantity. *N*_parasites_ at *t* = 0 and *N*_parasites_ at the end of the experiment (*t* = 48 h for the nine first suspensions and *t* = 96 h for the three last suspensions) showed a positive correlation for both DNA and RNA, but was only marginally significant for DNA. This observation was quite unexpected but can be explained by the rapid decrease of detected parasites (90% of decrease in 2 days). Whatever the initial amount of shed *B. ostreae* was, most parasites were no longer detected after 2 days spent outside the host, explaining why the correlation between *N*_parasites_ at the beginning and *N*_parasites_ at the end of experiment is so weak.

DNA detection does not allow distinguishing between viable and non‐viable cells whereas RNA detection could be a better proxy of metabolically active cells (Blais *et al.*, 1997; Klein and Juneja, 1997; McCarthy *et al.*, 2001; Nocker *et al.*, 2007; Bae and Wuertz, 2009; Pochon *et al.*, 2017). Nevertheless, it has been shown that eDNA persistence is lower in marine waters than in other systems, such as freshwater. Indeed, eDNA fragments persist above detection threshold for only few days in seawater and for several days or weeks in freshwater (Dejean *et al.*, 2011; Thomsen *et al.*, 2012; Sassoubre *et al.*, 2016; Collins *et al.*, 2018), making DNA detection in marine waters a good proxy for the presence of targeted organism. Thus, the combined DNA and RNA detection at a given time was considered as the best indicator of the presence of metabolically active parasites in this study. In our conditions, DNA detection appeared more repeatable than RNA detection with mean coefficients of variation estimated at 0.74 (±0.49) and 1.24 (±0.51) respectively. Therefore, although DNA detection does not inform on the cell status (viable or non‐viable), it still appears as an interesting target to monitor *B. ostreae* presence in seawater.

The use of both DNA and RNA detection approaches to monitor *B. ostreae* presence in seawater yielded consistent results. An increase in parasite detection was observed in the first hours post‐shedding (8 h for DNA and 28 h for RNA) and was followed by a slow decrease until the end of the experiment. Although unexpected, the increase in DNA and RNA amounts during the first hours post‐shedding was not statistically different from results at *t* = 0 (*P*‐value > 0.1, Wilcoxon–Mann–Whitney). Even if *B. ostreae* DNA and RNA could still be detected after 7 days, less than 10 % of the initial parasite amount remained detectable 48 h post‐shedding, suggesting that most of *B. ostreae* remain present for 2 days in seawater. This result is in agreement with previous work carried on this model (Arzul *et al.*, 2009).

Finally in tested conditions, more than 90% of shed parasites were no longer detected after 2 days. However, it was possible to detect *B. ostreae* DNA and RNA up to 7 and even 30 days respectively. The significance of these detections for transmission is unclear as detecting DNA and RNA does not inform on the capacity of the parasite to infect new oysters. Moreover, we can assume that the parasite presence can be impacted by environmental conditions including temperature and salinity (Arzul *et al.*, 2009) but also by other environmental factors such as light, turbidity or pH and the presence of other microorganisms in seawater.

By efficiently detecting and quantifying *B. ostreae* DNA and RNA in seawater, the methodology we have established can be used to monitor the presence of the parasite in zones with wild oyster beds and shellfish farms, but also to evaluate the effect of changing environmental factors on the detection and transmission of the parasite.

## Experimental procedures

### Selection of flat oysters infected with *Bonamia ostreae*

Flat oysters collected from *Bonamia ostreae* infected zones in Brittany (France) were sent to the laboratory and maintained in 225 l raceways supplied by natural seawater (15°C, 35 g NaCl l^−1^) enriched in *Skeletonema costatum* (10^10^ cells per hour) for at least five days before being biopsied. A piece of gill was collected from each oyster after maintaining them in a 50 g l^−1^ MgCl_2_ anaesthetic solution for at least 4 h (Suquet *et al.*, 2010). After drying tissues on absorbent paper, imprints were made on a glass slide and stained with Hémacolor^®^ (Merck‐Millipore). Gill imprints were screened for the presence of *B. ostreae* under light microscope and infection intensity was determined according to previously established criteria (Hervio *et al.*, 1995):

- Negative: No parasite detected in a whole imprint,
- Low infection (+): About 10 parasites observed in a whole imprint,
- Moderate infection (++): About one parasite per observation field,
- High infection (+++): Several or numerous parasites observed in each microscopic field.

Oysters found infected were selected either to isolate parasites and establish standard curves and detection limits of our methods or monitor the presence of the parasite in seawater.

### Real‐time PCR efficiency, limit of detection, limit of quantification and standard curves

Efficiency, LOD and LOQ of both real‐time PCR approaches were established using *Bonamia ostreae* isolated from highly infected oysters (Mialhe *et al.*, 1988). Isolated parasites were counted using a Malassez‐cell hemocytometer, and suspension concentration was adjusted before performing 1:10 serial dilutions in 0.22 µm filtered seawater (FSW). Each dilution was filtered onto a 1 µm pore size 47 mm diameter polycarbonate membrane (Whatman^®^ Nuclepore™ Track‐Etched Membranes), stored at −80°C until being used for DNA or RNA extraction. A total of 5 separate diluted series from 10 to 10^6^ parasites per membrane were tested in real‐time PCR for DNA detection. A total of 4 separate diluted series from 3 × 10^−1^ parasites to 3 × 10^7^ parasites per membrane were tested in real‐time PCR for RNA detection.

### Monitoring of *Bonamia ostreae* presence in seawater

The monitoring of *Bonamia ostreae* presence in seawater was studied using four batches of 7 to 10 flat oysters known to be infected with *B. ostreae* by examination of gill imprints as described above. Each batch included approximately 1/3 of lightly, 1/3 of moderately and 1/3 of highly infected oysters.

**Fig. 4:**
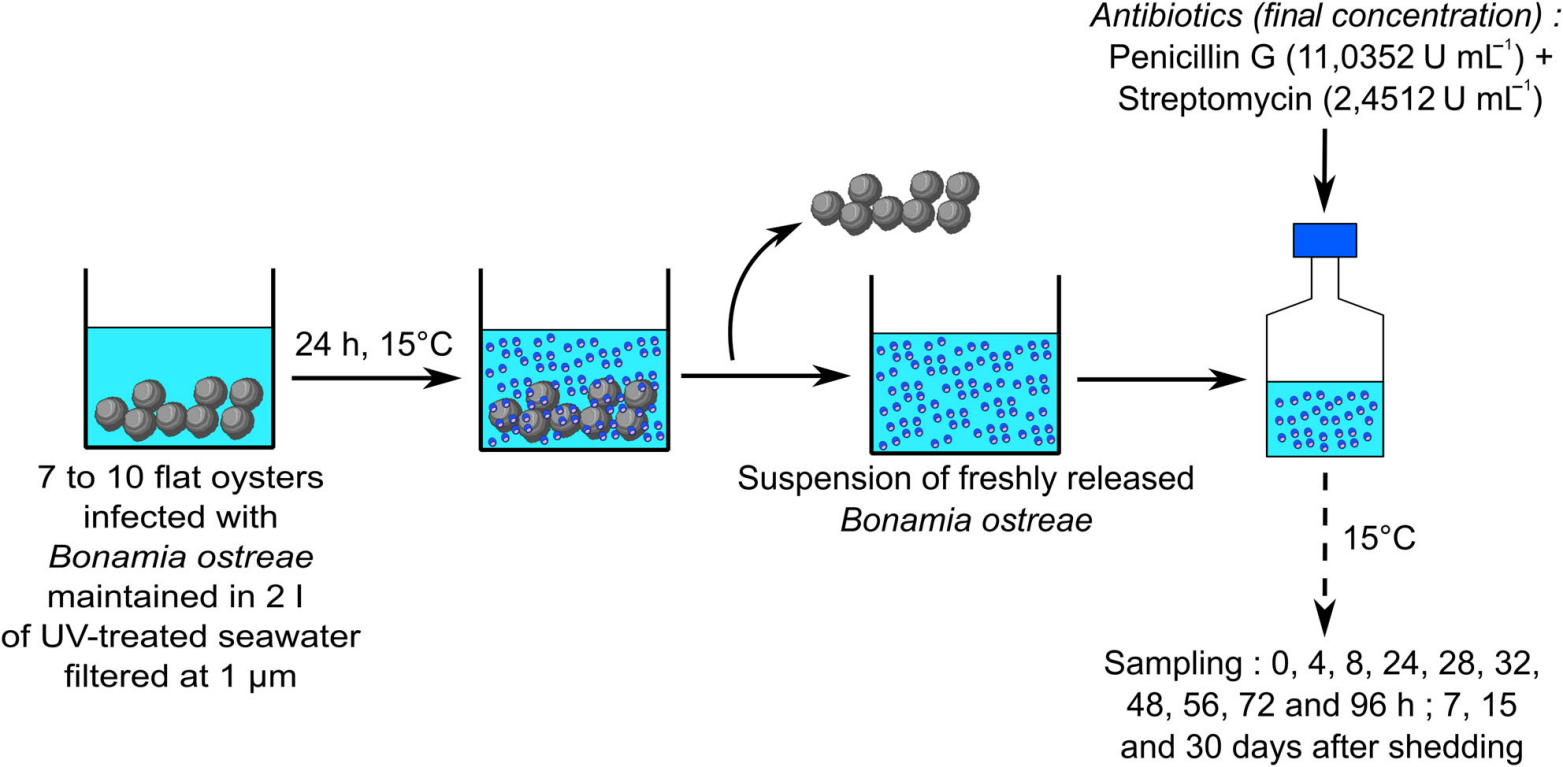
Experimental design used to monitor the presence of *Bonamia ostreae* outside flat oysters.

Oysters were maintained for 24 h in 2 l of UV‐treated seawater filtered at 1 µm. After 24 h, oysters were removed, seawater containing freshly released parasites was transferred into a 2 l glass bottle and complemented with streptomycin–penicillin G (final concentration: 2.4512 U ml^−1^ and 11.0353 U ml^−1^ respectively) to avoid bacteria proliferation. Nine parasite suspensions were obtained following this approach and were stored at 15°C and exposed to daylight. 100 ml of parasite suspension was collected after mixing suspension at days 0, 1, 2, 7, 15 and 30 (Fig. 4). Considering first obtained results, three additional parasite suspensions were prepared following the same approach to monitor parasite presence on a shorter period. In these trials, 100 ml of parasite suspension was collected after mixing suspension 0 h, 4 h, 8 h, 1 day, 28 h, 32 h, 2 days, 56 h, 3 days and 4 days after removing oysters (Fig. 4).

Negative controls consisted in 100 ml of seawater collected before immersing flat oysters.

### Sample filtration

Seawater samples (100 ml) collected as previously described were pre‐filtered at 20 µm to remove largest particles before being filtered through a 1 µm pore size 47 mm diameter polycarbonate membrane (WhatmanWhatman® Nuclepore™ Track‐Etched Membranes) using a vacuum pump. Each membrane was then cut in four quarters and stored at −80°C until being processed for either DNA (two quarters) or RNA (two quarters) extraction. Quarters for DNA extraction were stored in 1.5 ml Safe‐Lock Tubes (Eppendorf), and quarters for RNA extraction were stored in 1.5 ml DNA LoBind Tubes (Eppendorf). For each sample, one quarter membrane was used for nucleic acid extraction (DNA and RNA), and the other quarter was stored in order to redo analysis if needed.

### Nucleic acid extraction

Total DNA was extracted from quarter membranes using the DNeasy^®^ PowerWater^®^ Kit (Qiagen) according to the manufacturer’s protocol with some modifications. After 10 min at 65°C, a mechanical cell lysis was carried out using the Precellys^®^ 24 bead beater (Bertin Technologies) and the following programme: 4 lysis cycles of 20 s at 5000 r.p.m., with 5 s of pause between each cycle. DNA was eluted in 50 µl of the elution solution provided in the kit, and samples were stored at 4°C until being tested by real‐time PCR.

Total RNA was extracted from quarter membranes using the RNeasy^®^ PowerWater^®^ Kit (Qiagen) according to the manufacturer’s protocol with some modifications. Mechanical cell lysis was carried out as described above for DNA extraction. Nucleic acid was eluted in 50 µl of the elution solution provided in the kit. Genomic DNA was removed using the DNase™ Max Kit (Qiagen) following the manufacturer’s recommendations, except that DNase reaction was performed at 37°C for 30 min instead of 20 min. The successful elimination of genomic DNA was checked by testing RNA extracts directly by real‐time PCR targeting *Bonamia ostreae* 18S rDNA (see below).

cDNAs were synthesized using the Invitrogen™ SuperScript™ III Reverse Transcriptase kit (Thermo Fisher Scientific), following the manufacturer’s instructions. cDNAs were stored at 4°C until being tested by real‐time PCR.

### Real‐time PCR

For the detection of *Bonamia ostreae* 18S rDNA, amplification reactions were carried out as described in the Standard Operating Procedure (SOP) published by European Reference Laboratory for Mollusc Diseases (EURL for Molluscs Diseases, 2020; L. Canier, C. Dubreuil, M. Noyer, D. Serpin, B. Chollet, C. Garcia and I. Arzul, unpublished data). PCR mixture included 12.5 µl 2X Brilliant III Ultra‐Fast QPCR Master Mix (Agilent Technologies); 0.38 µl Bosp2 18S Forward Primer (5′ CAG GAT GCC CTT AGA TGC TC 3′) (20 µM); 0.63 µl Bosp2 18S Reverse Primer (5′ GTA CAA AGG GCA GGG ACG TA 3′) (20 µM); 0.75 µl Bosp2 18S IN Probe (5′ TTG ACC CGG CTT GAC AAG GC 3′) (HEX‐BHQ‐1) (10 µM); 5.75 µl bi‐distilled water; and 5 µl of extracted DNA. The thermal programme was as follows: 95°C for 3 min and 40 cycles of amplification at 95°C for 15 s and 60°C for 1 min.

For the detection of *B. ostreae* 18S rRNA, PCR mixture included 10 µl 2X Brilliant III Ultra‐Fast SYBR^®^ Green QPCR Master Mix (Agilent Technologies), 2 µl 18S Forward Primer (5′ TCA GCA CTT TTC GAG AAA TCA A 3′) (5 µM), 2 µl 18S Reverse Primer (5′ CCA CCA TGC ATA GAA TCA AGA A 3′) (4 µM), 1 µl bi‐distilled water and 5 µl of undiluted cDNA. The thermal profile was as follows: 95°C for 3 min and 40 cycles of amplification at 95°C for 5 s and 60°C for 20 s. A post‐PCR dissociation curve was run with the following parameters: 95°C for 1 min, 60°C for 30 s and a gradual augmentation from 60°C to 95°C for 40 min (Gervais *et al.*, 2018).

Each sample was analysed in duplicate by real‐time PCR analysis in 96‐microwell plates using the Mx3000p™ thermocycler sequence detector (Stratagene). Positive and negative controls were included in each PCR run. Positive controls consisted in DNA or RNA extracted from known infected samples. Negative controls consisting in 5 µl of bi‐distilled water used in the extraction and real‐time PCR steps were added to each PCR plate.

### Data analysis

Data were analysed using R 3.4.2 (2017‐09‐28) (R Core Team, 2017) after computing average *Ct* from each duplicate. When no amplification was obtained, ‘No Ct’ value was replaced by 40, corresponding to the maximum number of cycles.

#### Real‐time PCR efficiency, limit of detection and limit of quantification

Efficiency, LOD and LOQ of both DNA and RNA approaches were determined from the analysis of parasite dilution series in real‐time PCR.

After a decimal log conversion of the number of parasites included in the dilution series, PCR efficiency was established for each dilution series (*n* = 5 and *n* = 4 for DNA and RNA respectively) using the following formula:

(1) $$\text{Efficiency}\;\left(\%\right)=\left(10^{-1/a}-1\right)*100$$

where ‘*a*’ is the slope of the linear regression performed on *Ct* ~ log value.

Based on these data, average efficiency and standard deviation were computed for both DNA and RNA approaches.

To be as close as possible to the MIQE guidelines (Bustin *et al.*, 2009), LOD was defined as the lowest detected amount of parasites in at least 75% of tested dilutions (4 out of 5 for the DNA approach or 3 out of 4 for the RNA approach) and LOQ was defined as the last amount of parasites detected in the linear dynamic range of the standard curve.

#### Standard curves

Standard curves were then established for each real‐time PCR approach by performing a linear regression on semi‐logarithm transformed data obtained from the analysis of parasite dilution series. The equation of the standard curves was the following:

(2) $$Ct=\alpha \times \ln(N_{\text{parasites}})+\beta$$

95% confidence and prediction intervals were calculated using *predict* function in R (Cornillon *et al.*, 2012).

As an output of the regression model, *R* ^2^ was used to check the amount of variability of *Ct* explained by *N*_parasites_. Residuals were also analysed graphically to validate the model.

#### Parasite presence

Parasite presence was measured by testing samples regularly taken from freshly shed parasite suspensions in real‐time PCR.

Number of parasites per quarter membrane was calculated using the following equation (modified from Equation 2):

(3) $$N_{\text{parasites}}=e^{(Ct-\beta )/\alpha }$$

Parasite presence was then estimated at each time by computing the following ratio:

(4) $$(N_{\text{parasites},t=\tau }/N_{\text{parasites},t=0})\times 100$$

After testing data normality for each time, non‐parametric statistical tests (Wilcoxon–Mann–Whitney) were performed on presence data to investigate the difference between the amount of detected parasites at *t *= *τ* and at *t* = 0.

Additionally, correlation between the number of detected parasites at the beginning and the end of the experiment as well as between DNA and RNA detection was tested by computing Pearson’s, Spearman’s or Kendall’s correlation coefficients after checking data normality.

## Conflict of interest

The authors declare that they have no conflict of interest.
